# On the nature of picobirnaviruses

**DOI:** 10.18699/VJGB-23-32

**Published:** 2023-06

**Authors:** A.Yu. Kashnikov, N.V. Epifanova, N.A. Novikova

**Affiliations:** I.N. Blokhina Nizhny Novgorod Research Institute of Epidemiology and Microbiology, Nizhny Novgorod, Russia; I.N. Blokhina Nizhny Novgorod Research Institute of Epidemiology and Microbiology, Nizhny Novgorod, Russia; I.N. Blokhina Nizhny Novgorod Research Institute of Epidemiology and Microbiology, Nizhny Novgorod, Russia

**Keywords:** picobirnavirus, genome segment, host cell, mitochondrial genetic code, phylogenetic tree, reassortment, пикобирнавирус, сегмент генома, клетка-хозяин, митохондриальный генетический код, филогенетическое дерево, реассортация

## Abstract

The picobirnaviruses (Picobirnaviridae, Picobirnavirus, PBVs) are currently thought to be animal viruses, as they are usually found in animal stool samples. However, no animal model or cell culture for their propagation has yet been found. In 2018, a hypothetical assumption about PBVs belonging to prokaryotic viruses was put forward and experimentally substantiated. This hypothesis is based on the presence of Shine–Dalgarno sequences in the genome of all PBVs before three reading frames (ORF) at the ribosomal binding site, with which the prokaryotic genome is saturated, while in the eukaryotic genome such regions occur with low frequency. The genome saturation with the Shine–Dalgarno sequences, as well as the preservation of this saturation in the progeny, according to scientists, allows us to attribute PBVs to prokaryotic viruses. On the other hand, there is a possibility that PBVs belong to viruses of eukaryotic hosts – fungi or invertebrates, since PBV-like sequences similar to the genome of fungal viruses from the families of mitoviruses and partitiviruses have been identified. In this regard, the idea arose that, in terms of reproduction mode, PBVs resemble fungal viruses. The divergence of views on the true PBV host(s) has sparked discussions among scientists and required further research to elucidate their nature. The review highlights the results of the search for a PBV host. The reasons for the occurrence of atypical sequences among the PBV genome sequences that use an alternative mitochondrial code of lower eukaryotes (fungi and invertebrates) for the translation of viral RNA-dependent RNA polymerase (RdRp) instead of the standard genetic code are analyzed. The purpose of the review was to collect arguments in support of the hypothesis about the phage nature of PBVs and to find the most realistic explanation of the reasons for identifying non-standard genomic sequences for PBVs. Based on the hypothesis about the genealogical relationship of PBVs with RNA viruses from other families with similar segmented genomes, such as Reoviridae, Cystoviridae, Totiviridae and Partitiviridae, virologists support the assumption of a decisive role in the origin of atypical PBV-like reassortment strains between PBVs and viruses of the listed families. The collected arguments given in this review indicate a high probability of a phage nature of PBVs. The data presented in the review show that the belonging of PBV-like progeny to prokaryotic or eukaryotic viruses is determined not only by its genome saturation level with a prokaryotic motif, standard or mitochondrial genetic code. The primary structure of the gene encoding the viral capsid protein responsible for the presence or absence of specific proteolytic properties of the virus that determine its ability for independent horizontal transmission into new cells may also be a decisive factor.

## Abstract (ru)

Считается, что пикобирнавирусы (Picobirnaviridae, Picobirnavirus, ПБВ) являются вирусами животных, так как их обычно находят в образцах стула животных. Однако до сих пор не найдена модель животного или клеточная культура для их размножения. В 2018 г. было выдвинуто и экспериментально обосновано гипотетическое предположение о принадлежности ПБВ к вирусам прокариот. Эта гипотеза основана на присутствии в геноме всех ПБВ перед тремя открытыми рамками считывания (ORF) в сайте связывания с рибосомой последовательностей Шайна–Дальгарно, которыми насыщен геном прокариот, тогда как в геноме эукариот такие участки встречаются с низкой частотой. Насыщенность генома последовательностями Шайна–Дальгарно, а также сохранение такой насыщенности у потомства, по мнению ученых, позволяет отнести ПБВ к вирусам прокариот. В то же время существует вероятность принадлежности ПБВ к вирусам эукариотических хозяев – грибов или беспозвоночных, поскольку были обнаружены ПБВ-подобные последовательности, сходные с геномом вирусов грибов из семейств митовирусов и партитивирусов. В связи с этим возникло представление, что по способу репродукции ПБВ напоминают вирусы грибов. Расхождение во взглядах на истинного хозяина (хозяев) ПБВ вызвало дискуссию среди ученых и потребовало дальнейших исследований для выяснения их природы В обзоре освещены результаты исследований по поиску хозяина ПБВ. Проанализированы причины появления среди множества характерных для ПБВ последовательностей генома атипичных последовательностей, использующих для трансляции вирусной РНК-зависимой РНК-полимеразы (RdRp) вместо стандартного генетического кода альтернативный митохондриальный код низших эукариот (грибов и беспозвоночных). Цель обзора состояла в сборе аргументов в поддержку гипотезы, полагающей фаговую природу ПБВ и поиск наиболее реалистичного объяснения причин выявления нестандартных для ПБВ геномных последовательностей. Опираясь на гипотезу о генеалогическом родстве ПБВ с РНК-вирусами из других семейств со сходным сегментированным геномом, такими как Reoviridae, Cystoviridae, Totiviridae и Partitiviridae, вирусологи поддерживают предположение о решающей роли в происхождении атипичных ПБВ-подобных штаммов реассортации между ПБВ и вирусами перечисленных семейств. Собранные аргументы свидетельствуют о большой вероятности фаговой природы у ПБВ. Представленные в обзоре данные свидетельствуют, что принадлежность ПБВ-подобного потомства к вирусам прокариот или эукариот определяется не только степенью насыщения его генома прокариотическим мотивом, стандартным или митохондриальным генетическим кодом. Решающим фактором может являться также первичная структура гена, кодирующего белок вирусного капсида, отвечающего за наличие или отсутствие специфических протеолитических свойств у вируса, определяющих его способность к самостоятельному горизонтальному распространению в новые клетки

## Introduction

Picobirnaviruses (PBVs) are small, nonenveloped bisegmented double-stranded RNA viruses that have been detected in a wide variety of animal species including invertebrates and in environmental samples. Since PBVs are ubiquitous in faeces/ gut contents of humans and other animals with or without diarrhea, they were considered opportunistic enteric pathogens of mammals and avian species. However, an animal cell culture or a gnotobiotic animal for propagation of this virus has not yet been identified. This fact led some researchers to doubt that picobirnaviruses belong to eukaryotic viruses. Indian scientists Krishnamurthy, Wang (2018) have analyzed a large number of full-size (almost full-size) genomic sequences of PBVs found in faeces of humans and animals as well as environmental samples. This analysis revealed prokaryotic motifs (regions) in the PBV genome located before the open reading frames at the ribosomal binding site. Based on the data obtained, a hypothesis was put forward and experimentally substantiated that PBVs belong to prokaryotic viruses – bacteriophages (Krishnamurthy, Wang, 2018), which was later supported by a number of other studies (Adriaenssens et al., 2018; Boros et al., 2018; Kleymann et al., 2020).

On the other hand, after the discovery of new PBV-like nucleotide sequences encoding RNA-dependent RNA polymerase, but using an alternative (non-standard) mitochondrial genetic code (of molds or invertebrates) for translation, it was suggested that PBVs could be fungal viruses with a reproduction mode resembling that of mitoviruses (Yinda et al., 2019; Kleymann et al., 2020).

These contradictions, which have caused a discussion in the scientific community on the nature of the true host(s) of PBVs, can be resolved by a hypothesis put forward in 2018 by Wolf et al. (2018) and supported by other researchers (Chauhan et al., 2021). This hypothesis explains the origin of abnormal strains of PBVs by the tendency of these viruses to abrupt genetic modification following the reassortment of genome segments described earlier (Conceição-Neto et al., 2016) and the acquisition of the ability of the bacteriophage to reproduce in the cells of the organism of another taxonomic group – the lower eukaryote.

This review analyzes the available publications on modern ideas about the origin and evolution of PBVs, as well as a discussion on the prokaryotic or eukaryotic nature of their true hosts. The purpose of the review was to collect arguments to support the hypothesis about the phage nature of PBVs and to search for the most realistic explanation of the reasons for the identification of genomic sequences that are nonstandard for viruses.

## Characteristics of PBVs

**Fig. 1.**
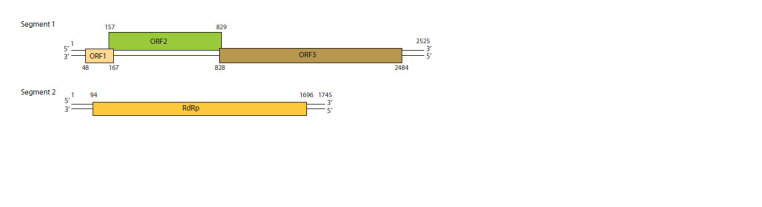
Genomic organization of the human PBV (strain Hy005102) belonging to the genogroup I (Ghosh, Malik, 2021). Segment 1 of Hy005102 genome consists of three open reading frames (ORF) ORF1, ORF2, ORF3. Reading frame ORF3 encodes the precursor of the virus capsid protein. Segment 2 has one ORF encoding viral RNA-dependent RNA polymerase (RdRp). ORF1 and ORF2 products are not identified

Picobirnaviruses (PBVs) are small, noneveloped particles 33–37 nm in diameter with icosahedral type of symmetry (T = 2) belonging to the only genus Picobirnavirus within the family Picobirnaviridae of the order Diplohnavirales. Double-stranded (ds) RNA-genome of PBVs consists of two segments 2525 and 1745 bp in length (Fig. 1). Information about the structure of the virion and genome of PBV, the area of prevalence, connection with diarrhea, the level of excretion, the opportunistic (conditionally pathogenic) and zoonotic nature of the virus, the wide tissue tropism and genetic variability is given in previously published reviews (Ganesh et al., 2014; Kashnikov et al., 2020; Ghosh, Malik, 2021). The methods of amplification, PCR diagnostics and genome sequencing of these viruses are also described there

**Fig. 2.**
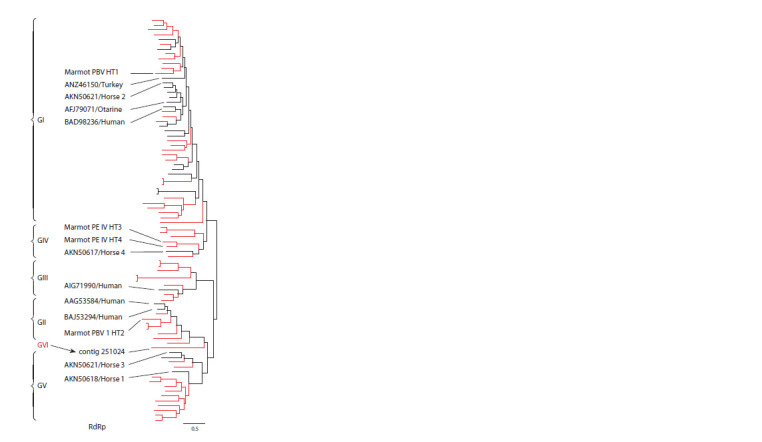
Phylogenetic tree showing the presence of five proposed PBV genogroups identified in marmot is built on the basis of the nucleotide sequences of the complete RdRp gene. Sequences of picobirnaviruses obtained from marmot are shown in red. Sequences of picobirnaviruses obtained from other hosts are shown in black (Luo et al., 2018, with modifications).

Using phylogenetic analysis based on the nucleotide sequence of the RNA-dependent RNA polymerase (RdRp) gene located in the segment 2 of the genome, researchers divide PBVs into five genogroups: GI, GII (Rosen et al., 2000), GIII (Smits et al., 2014), GIV and GV (Li et al., 2015), among which there are genetically variable clusters. Genogroups GI and GII are more common in the PBV cluster detected in vertebrates and humans. Genogroup GIII has been identified in invertebrates (Shi et al., 2016) while genogroups GIV and GV have been identified in fungal and prokaryotic host cells (Knox et al., 2018). All five PBV genogroups identified in one host (marmot) are shown in the phylogram (Fig. 2) in the study (Luo et al., 2018). The main PBV genogroups are genogroups GI and GII, of which PBVs of genogroup GI are the most common (Shi et al., 2016; Kumar et al., 2020; Ghosh, Malik, 2021).

It has been established that viruses belonging to the same RdRp-genogroup can be detected in suspected hosts belonging to different species. It has also been shown that PBVs of different genogroups are detected in the host of the same species (Ganesh et al., 2014; Malik et al., 2014; Woo et al., 2014, 2019; Li et al., 2015; Gallagher et al., 2017; Navarro et al., 2017; Boros et al., 2018; Duraisamy et al., 2018; Ghosh et al., 2018; Yinda et al., 2019; Joycelyn et al., 2020; Kleymann et al., 2020; Ghosh, Malik, 2021). At the same time, the true host of viruses has not yet been identified. Among the higher eukaryotes, they did not succeed in identifying either a cell culture or gnotobiotic animals for the virus propagation (Ganesh et al., 2014; Malik et al., 2014; Delmas et al., 2019; Kleymann et al., 2020).

In the future, as the PBV studies continued, researchers began to doubt the fact that the cells of higher eukaryotes could be the hosts of these viruses (Adriaenssens et al., 2018; Boros et al., 2018; Krishnamurthy, Wang, 2018). Recently it has been discovered that PBVs differ from dsRNA viruses of higher eukaryotes (Reoviridae) not only in the architecture of the capsid, but also in presumably being able to infect prokaryotic cells (Knox et al., 2018; Krishnamurthy, Wang, 2018).

## Hypothesis about the phage nature of PBV and associated doubts

Prior to the hypothesis of Krishnamurthy and Wang (2018), PBVs were thought to be eukaryotic viruses because they were identified in a wide variety of animal species, including invertebrates. Since PBVs are ubiquitous in the gut contents of humans and other animals with or without diarrhea, they were considered opportunistic enteric pathogens. But intestinal virom in animals contains not only eukaryotic, but also prokaryotic viruses, which usually make up the largest share of it (Yinda et al., 2018). It was logical to assume that PBVs are not present in the gut cells, but in the gut contents and can be prokaryotic viruses of the gut microbiome (Adriaenssens et al., 2018; Kunz et al., 2018; Delmas et al., 2019; Bell et al., 2020; Guajardo-Leiva et al., 2020; Ghosh, Malik, 2021).

In this case, the level of virus identification should correspond to the number of bacteria in which they multiply. In particular, the study of Kleymann et al. (2020) reported high rates of identification of GI PBVs (35.36 %, 29/82) in Indian mongoose stool samples on the island of St. Kitts (one of the Lesser Antilles). The percentage of PBV identification on the island of St. Kitts could mean the concentration of host bacteria in this area. Moreover, there was a difference in the frequency of PBV identification in mongooses from the urban and wild habitats, 33.33 % (19/57) and 40.00 % (10/25) respectively, which could indicate a difference in bacterial load in these places

The suggestion that the PBVs may be prokaryotic viruses appeared when researchers began to analyze the diversity of full-sized (almost full-sized) sequences of their genome. With the help of next-generation sequencing technologies (NGS) and polymerase chain reaction (PCR) using specific primers, they succeeded in identifying some features of the PBV genome that indicate that these viruses may actually be prokaryotic or fungal viruses (Shi et al., 2016; Adriaenssens et al., 2018; Boros et al., 2018; Krishnamurthy, Wang, 2018; Wolf et al., 2018; Yinda et al., 2018; Delmas et al., 2019; Kleymann et al., 2020; Ghosh, Malik, 2021)

In 2018, while analyzing different reference genomes of RNA-containing viruses individually and at the family level, Indian scientists Krishnamurthy and Wang have discovered conservative regions in the PBV genome called Shine–Dalgarno sequences or SD-sequences (Krishnamurthy, Wang, 2018). Such regions are present in the genomes of all prokaryotic and eukaryotic viruses and usually consist of six nucleotides – AGGAGG. They are located in the 5′-untranslated region before the open reading frames (ORFs) at a distance of 1 to 18 nucleotides (spacer region) to the start codon (AUG) initiating the translation of the viral genome products (Krishnamurthy, Wang, 2018; Ghosh, Malik, 2021). Functional SD-sequences are ribosome binding sites and promote the translation of viral proteins.

However, genome enrichment with SD-sequences was observed only in families of viruses that infect prokaryotes, but not in families infecting eukaryotes. This observation made it possible for Krishnamurthy and Wang to suggest that the high frequency of appearance of SD-sequences in the viral genomes may be a defining feature of the prokaryotic type of virus, and any viral families the genomes of which are enriched with such SD-regions are prokaryotic viral families. Among the viruses infecting prokaryots, for example, some bacteriophages of the family Cystoviridae have a high content of SD-sequences, the genome of which consists of several fragments of dsRNA (Mindich, 1988; Boros et al., 2018).

In the PBV genome, SD-sequences were present before all ORFs in segments 1 and 2. The level of enrichment with SDregions in PBVs is higher than in any known prokaryotic virus family and this level was constant (in 100 % of the studied genes), while not in all prokaryotic viruses it is maintained in the virus replication process. Such a high level of preservation of prokaryotic regions in the PBV genome should correlate with the level of their preservation in the genome of bacteria of a certain type from the spectrum of hosts that PBVs can infect. This level varies in different viral families (Krishnamurthy, Wang, 2018). Preservation of the level of enrichment with prokaryotic regions in the genomes of prokaryotic viruses depends on whether the host bacterium itself retains them in its genes. It is known that not all bacterial species preserve the prokaryotic Shine–Dalgarno sequence to the same extent. For example, in the genome of bacteria belonging to the type Firmicutes, the prokaryotic motif is preserved in more than 80 % of genes, while in Bacteroides, less than 10 % (Omotajo et al., 2015). It is known that different families of bacterial RNA-viruses can consist of evolutionarily related viruses capable of infecting one type of bacteria. From this fact it follows that PBVs can infect bacteria within the type Firmicutes that most corresponds to the level of preservation of the prokaryotic motif in genes (more than 80 %) to PBVs and is most common in the fecal microbiota (Sekelja et al., 2011).

The hypothesis about the phage nature of PBVs put forward by Krishnamurthy and Wang (2018) is confirmed by the results obtained by other researchers (Adriaenssens et al., 2018; Boros et al., 2018; Kleymann et al., 2020). In particular, the study of Boros et al. (2018) revealed in the genome of chicken PBVs SD-regions that were located in segment 1 before the three ORFs and in segment 2 before ORFs above the initiation codons. Using 6xHis tagging and western blotting of genomic segment 1 of PBVs containing the SD-motif, these researchers have succeeded in showing in vivo the possibility of its expression and functionalization in Escherichia coli (Boros et al., 2018). The results obtained, according to the authors, serve as proof of the existence of a bacterial culture for the reproduction of PBVs.

The assumption that PBVs represent a new family of RNA bacteriophages with a high level of genomic diversity was also confirmed in the work of Adriaenssens et al. (2018). The authors of this work found a hexameric prokaryotic AGGAGG motif in 100 % of the genomic sequences of PBVs, while eukaryotic viruses from different families had SD-regions with low frequency and mainly consisted of tetramers (AGGA, GGAG, GAGG) (Adriaenssens et al., 2018). The review of Ghosh, Malik (2021) presents a number of conservative prokaryotic sequences (motives) found before all ORFs in segments 1 and 2 of PBV and PBV-like genomes, indicating the location and with access numbers in GenBank (Ghosh, Malik, 2021).

However, despite the practical results obtained, indicating the possible PBV affiliation to prokaryotic viruses, many authors believe that it is premature to talk about the final proof of the phage nature of PBVs (Ramesh et al., 2021). The host in which PBVs would successfully reproduce has not yet been identified. Given the fact that the gut microbiome consists of several hundreds of mostly uncultivable bacteria, the identification of true bacterial or archeal host(s) of PBVs (if any) will be challenging (Boros et al., 2018).

In addition, ORFs encoding the RdRp gene were found in the genome of some PBV strains, in which, during translation, instead of the expected standard genetic code, an alternative code of fungi and invertebrates was used (Shi et al., 2016; Yinda et al., 2018, 2019; Kleymann et al., 2020). So an assumption was made that the PBV hosts may be cells of lower eukaryotes.

## PBV-like strains with nonstandard genetic code

As is known, the gene sequences in the viral genomes have their characteristic conserved regions – motifs by which viruses are identified. Motives characteristic of PBV and PBVlike genomes include the prokaryotic region Shine– Dalgar- no AGGAGG (Adriaenssens et al., 2018; Boros et al., 2018; Ghosh et al., 2018; Krishnamurthy, Wang, 2018; Yinda et al., 2018, 2019; Guajardo-Leiva et al., 2020; Joycelyn et al., 2020; Kleymann et al., 2020), terminal sequences 5′-GUAAA and 3′-ACUGC (Ghosh et al., 2018; Delmas et al., 2019; Woo et al., 2019; Kleymann et al., 2020), and three regions represented in amino acid sequences DFXKFD, SGSGGT and GDD (Kleymann et al., 2020)

**Fig. 3.**
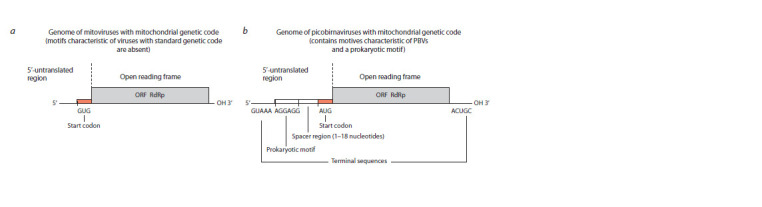
Mitoviruse (a) and picobirnaviruse (b ) genomes.

When translating the RdRp gene of most picobirnaviruses, the standard genetic code is used. However, recently, new PBV-like RdRp gene sequences with an alternative (nonstandard) mitochondrial genetic code have been identified in human (Woo et al., 2019), mongoose (Kleymann et al., 2020), bat (Yinda et al., 2018) and invertebrate (Shi et al., 2016) fecal samples. The mitochondrial code is characteristic of viruses of mold fungi and invertebrates. In particular, five PBV-like genomic sequences consisting of dsRNA with mitochondrial code were isolated from the gut contents of bats (Yinda et al., 2018). In four of them (P11-300, P11-378, P14-90 and P15- 218) they failed to identify the presence of the capsid gene. These PBV-like genomes contained only RdRp gene sequence with the mitochondrial genetic code of the mold. The absence of the capsid protein gene in them resembled the genome of mitoviruses from the family Mitoviridae, which are known to infect the mitochondria of unicellular mold fungi (Fig. 3) (Hillman, Cai, 2013; Shi et al., 2016).

The mitovirus genome as well as four abnormal PBVlike genomes consists of dsRNA and encodes only RdRp. Mitoviruses lack a capsid, replicate in mitochondria and use the genetic mitochondrial code of mold fungi for the RdRp translation. So an assumption was made that mold fungi can also be PBV hosts (Yinda et al., 2018; Kleymann et al., 2020).

However, unlike mitoviruses, the noncapsid PBV-like strains identified by Yinda et al. (2018) in a single segment of the RdRp genome contained conserved regions characteristic of PBVs. In the genome of the fifth PBV-like strain (P16-366), in addition to the conservative features of the RdRp gene characteristic of PBVs, a sequence encoding the capsid protein was found. This strain was clustered on the phylogenetic tree along with GII PBVs. However, it used an alternative genetic code and was very similar in terms of genome organization to fungal viruses of the family Partitiviridae (Duquerroy et al., 2009). These families have a minimalistic genome consisting of two dsRNA segments encoding RdRp and the capsid protein, respectively, which in these families is clearly homologous (Wolf et al., 2018).

Similarly, researchers Kleymann et al. (2020) isolated the M17A strain among typical PBV-like strains from mongoose, the RdRp gene of which retained all conservative for PBVs motives, but had an alternative mitochondrial mold code, and the capsid sequence in the genome of this strain was absent. Assumption that PBVs can be fungal or invertebrate viruses, as Ghosh, Malik (2021) rightly noted, has further complicated the discussion about the true PBV host

## Discussion on the origin of abnormal PBV-like strains

During the discussion about the true PBV hosts, researchers attempted to interpret the causes of the appearance of PBV-like strains (Shackelton et al., 2008; Wolf et al., 2018; Yinda et al., 2018; Shahi et al., 2019; Ghosh, Malik, 2021). In particular, Yinda et al. (2018) suggested that PBV-like strains found in the gut contents of different eukaryotic hosts, with a genome resembling the mitovirus genome, may have a reproduction mode similar to mitoviruses. Following the assumption of Yinda et al. (2018), the capsid protein gene is not needed with this reproduction mode, since mitoviruses do not use the pathway of the independent horizontal transmission from cell to cell, but are transmitted vertically from mother to daughter cells (during division) or horizontally (by merging hyphae). Similarly, when assembling new PBV-like particles, simplified structures can be formed with the adaptation to the existence in a fungal cell characteristic of mitoviruses and lost capsid protein gene. Such an interpretation of the appearance of noncapsid RNA viruses is consistent with one of the trends in the evolution of RNA viruses associated with the loss of their structural module by their genome, noticed by Wolf et al. (2018).

**Fig. 4.**
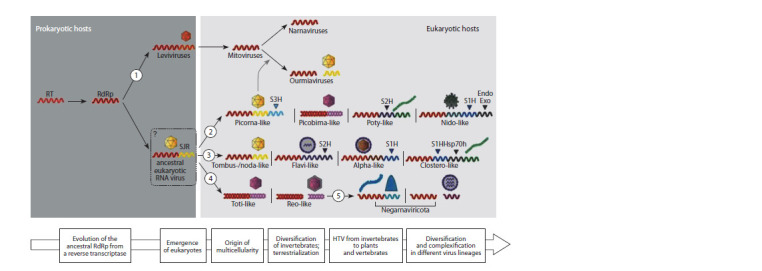
A rough scheme of the key steps of RNA virus evolution (Wolf et al., 2018).

The trend in the evolution of RNA viruses associated with the loss of the genome segment with the capsid gene is demonstrated by the pedigree diagram of eukaryotic viruses with the RNA genome, shown in Figure 4. This scheme also explains the origin of capsidated RNA viruses of unicellular eukaryotes

The evolution of capsid-free viruses of lower eukaryotes (fungi and invertebrates) follows the path of genome reduction due to the loss of the capsid protein gene of the probable ancestor – levivirus infecting prokaryotes (the vector of evolution under the number 1 in Figure 4, leading to the appearance at the end of the family Narvaviridae). In accordance with the scheme, the ancestor of mitoviruses from the family Mitoviridae descended from the family Leviviridae, previously containing the capsid protein gene. An illustrative example of genome reduction is hypoviruses – capsid-free viruses with dsRNA, the structure and phylogenetic analysis of the genome of which showed that they originated from potiviruses that lost the capsid protein (Dawe, Nuss, 2013; Chauhan et al., 2021) and the capsid protein gene, entering the cells of unicellular eukaryotes. Replication of mitoviruses occurs only in mitochondria, where their capsid-free (‘naked’) RNA genomes (replicons) are replicated. With the loss of the structural gene, mitoviruses have lost the ability for independent horizontal transmission (Shackelton, Holmes, 2008).

Similarly, the origin of the group of capsid-free PBV-like strains isolated by Yinda et al. (2018) and Kleymann et al. (2020) from Cameroonian bats (P11-300, P11-378, P14-90 and P15-218) and from a mongoose (strain M17A) can be explained by the loss of a genome segment encoding the capsid protein by the parent strain

On the other hand, a capsid-free RNA virus (mitovirus) in the process of evolution could gain a capsid (the final direction of vector 1, leading to the occurrence of the family Ourmiaviridae due to the fusion of its ‘naked’ RNA replicon with the replicon of the capsid protein of +eukaryotic RNA virus, possibly having the eukaryotic virus as an ancestor (evolution vector 2 in Figure 4). Encapsulated strains of RNA viruses, and probably PBV-like analogues, could have occurred in this evolutionary way.

Researchers explain shuffling or loss of genome segments in RNA viruses by another trend in their evolution – the ability to reassort – to redistribute gene modules (RdRp and capsid protein) between closely related virus families with similar genes. Reassortment modification of the genome has also been observed in PBVs (Conceição-Neto et al., 2016). The creative role of reassortment between families of RNA viruses with similar genes explains the origin of the PBVlike strain P16-366 found by Yinda et al. (2018) in bats. This strain contained, together with the gene sequence RdRp (with a non-standard mitochondrial code), the sequence encoding the capsid protein. The proposed genetic similarity between families of viruses with a bisegmented dsRNA genome is based on the hypothesis of a reassortment mechanism for the evolution of these viruses

## On the relationship of PBVs with families of viruses with the dsRNA genome

The phylogenetic relationship between the families of RNA viruses with a segmented genome is based on the information about the primary structure of the RdRp gene. This gene is universal and, despite a much greater distribution among eukaryotic viruses, is present in almost all RNA viruses (including capsid-less RNA replicons) with the exception of some satellite viruses (Dolja, Koonin, 2018).

**Fig. 5.**
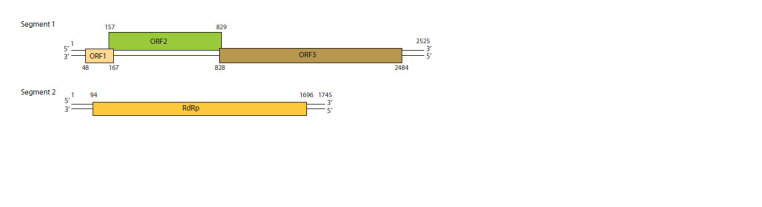
Phylogenetic tree of viruses with the RNA-genome built on the basis of gene sequences of RNA-dependent RNA polymerase and reverse transcriptase (Wolf et al., 2018). Five main branches are shown, among which three branches 1, 2 and 4 directly relate to the origin of PBV-like strains.

The universality of the RdRp gene indicates the possibility of its common origin in RNA viruses. In 2018, Wolf et al. put forward a hypothesis suggesting the presence of phylogenetic relationships between families of RNA-containing viruses, and a phylogenetic tree was constructed (Fig. 5), the topology of which demonstrates a possible relationship between the families

The phylogenetic tree constructed on the basis of a set of phylogenetic data gives an idea of the possible origin of PBVs. This tree confirms the evolution scenario in which the last common ancestors of virus lineages with a dsRNA genome were simple viruses the segmented genome of which contained two genes (RdRp and capsid protein). According to the authors of the hypothesis, all viruses with a dsRNA genome in branches 2 and 4 of the phylogenetic tree have similar capsid proteins that can be combined with the genome RdRp from different viruses with +RNA genome

Viruses with the dsRNA genome – Partitiviridae, Picobirnaviridae, Cystoviridae, Reoviridae and Totiviridae form separate lineages in two branches of the phylogenetic tree. Viruses of the family Picobirnaviridae are located on the same line with the families Hypoviridae, Amalgaviridae and Partitiviridae. The most phylogenetically close families forming one cluster are Picobirnaviridae and Partitiviridae. The close location of these families demonstrates a high degree of relationship between them. These families are united by a similar organization of virions and homologous capsid proteins (Duquerroy et al., 2009; Wolf et al., 2018). The difference between these viruses lies in the fact that the surface PBV capsid proteins, unlike those of partiviruses, have perforation activity, which determines the ability of the virus to penetrate into the cell. In addition, due to the ability of PBVs for horizontal transmission, two segments of its genome are combined into one capsid during assembly, and in partiviruses, the RdRp gene and the capsid protein gene use separate capsids (Vainio et al., 2018).

The PBV capsid protein gene is distantly related to the capsid protein genes of other viruses with the dsRNA genome (Totiviridae, Reoviridae and Cystoviridae), which make up three parallel evolutionary lineages in branch 4 of the phylogenetic tree (Wolf et al., 2018). Significant homology of the genes encoding the capsid protein of viruses of the families Reoviridae, Totiviridae, Cystoviridae, Picobirnaviridae and Partitiviridae was noted earlier (El Omari et al., 2013; Lvov et al., 2013; Luque et al., 2014).

Three evolutionary lineages of branch 4 of the phylogenetic tree are formed by families of viruses infecting both prokaryotes (Cystoviridae) and eukaryotes (Reoviridae, Totiviridae). The location of these viral families on one branch, according to the authors of the phylogenetic tree, does not exclude the possibility of the origin of eukaryotic +RNA viruses from their prokaryotic analogues. They admit that mitoviruses (replicating in the mitochondria of mold cells) originated from an ancestor common with leviviruses – a prokaryotic RNA virus parasitizing in enterobacteria. The proof of this is the evolutionary relationship between cystoviruses (bacterial viruses) and reoviruses (Poranen, Bamford, 2012; El Omari et al., 2013).

According to supporters of the creative role of reassortment, the origin of the PBV-like strain P16-366 could result from a reassortment between an as yet undiscovered PBV relative from branch 2, which has passed to reproduction in the mitochondria of fungal cells, and one of the viruses with the dsRNA genome of branch 4 (see Fig. 5). Moreover, it was noted that genetic restrictions on the ability to create reassortants during coinfection with viruses of families forming branch 4 of the phylogenetic tree may be less strict for the prokaryotic virus family Cystoviridae. The appearance of the encapsulated PBV-like strain P16-366 occurred due to the unification of the segment with the gene RdRp of the +RNA virus of branch 2 (possibly a naked RNA replicon) using the mitochondrial code, with a fragment of the capsid gene of the dsRNA virus of branch 4.

Another direction in the evolution of viruses with a bisegmented dsRNA genome is the acquisition of partition – the packaging of genome segments into one (monopartite) or separate (bi-multipartite) particles. This trend is interesting because it gives us some insight into the nature of the hosts of PBV-like strains (prokaryotic or eukaryotic). For example, bi-partition is observed only in fungal viruses with a dsRNA genome and in plant viruses with a ssDNA genome (Begomoviruses) (Nibert et al., 2013). In bacterial viruses in general and with a segmented dsRNA genome, in particular (Cystoviridae), the packaging of genome segments into individual particles is not observed. Partition is related to the virus transmission mode (independent or non-independent). For example, partitiviruses parasitizing in fungal cells do not have the ability for independent (horizontal) transmission and they are bipartite, while PBVs transmitting independently are monopartite. The horizontal (independent) transmission pathway allows them to transfer both segments of the genome into a new cell with a high probability. The ability for independent (horizontal) transmission into new cells can be considered as one of the main criteria for determining the true host of the detected virus.

## Presence of a molecular apparatus for penetration into the cell is an important criterion determining the nature of the PBV host

During familiarization with the studies solving the question of the nature of PBV and PBV-like strains, we came to understand that the belonging of these viruses to the bacterial viruses, higher or lower eukaryotes can be determined not only by the characteristic saturation level of their genome with a prokaryotic motif, standard or mitochondrial genetic code. This affiliation should no less be determined by the presence or absence of specific (in relation to the host cell) proteolytic activity of the capsid protein, which determines the possibility of independent penetration of the virus into the cell. The ability for independent horizontal transmission is characteristic of animal viruses and phages, while it is absent in viruses of lower eukaryotes (partitiviruses, mitoviruses). PBV capsid proteins have perforating activity (Duquerroy et al., 2009). This makes PBVs capable of independent penetration into cells and, therefore, can characterize them as bacterial viruses.

Thus, we can conclude that in solving the question of the true PBV host in which they can reproduce, in addition to conservative motifs characteristic of the PBV genome, the determining factor is the presence of a mechanism of specific horizontal penetration into the cell – capsid proteins with specific perforating activity. If these proteins are specific to the receptors of a prokaryotic cell, for example, Firmicutes cells (Adriaenssens et al., 2018), then we are dealing with a phage, if they are specific to the receptors of a eukaryotic cell – with a virus of higher eukaryotes, and if there are no capsid proteins at all, then such a virus can be attributed to viruses of lower eukaryotes (not capable of independent transmission).

## Assumption about the origin of atypical PBV-like strains does not contradict the hypothesis of the phage nature of PBVs

The existence of atypical PBV-like strains cannot constitute a refutation of supposed phage nature of PBVs for a number of reasons. According to the hypothesis of Wolf et al. (2018), there is a related relationship between the families of viruses with the dsRNA genome in the structure of the phylogenetic tree, as evidenced by the presence of homologous genes and a common ancestor related to prokaryotic RNA viruses. Moreover, the families of eukaryotic and prokaryotic RNA viruses may be related, as evidenced by the evolutionary relationship between cystoviruses and reoviruses (Poranen, Bamford, 2012; El Omari et al., 2013). This supports the assumption of the possibility of the exchange of homologous segments of the genome between prokaryotic viruses and related viruses of lower eukaryotes, when both are in the mold fungus cell.

It has recently become known that bacterial viruses can not only infect bacterial cells, but also pass through the epithelial cells of eukaryotes using the mechanism of phage transcytosis. From epithelial cells through the blood or lymph, phages can enter various organs and tissues of animals. However, phages can penetrate inside eukaryotic cell only with a bacterial cell infected by them (Nguyen et al., 2017). The ubiquitous isolation of PBVs from environments where bacteria occur means that these viruses may not be intracellular eukaryotic viruses, but prokaryotic viruses of the gut microbiome (Kashnikov et al., 2020; Ghosh, Malik, 2021). The assumption that PBVs can infect prokaryotic cells is confirmed by the presence of a conservative prokaryotic Shine–Dalgarno sequence in their genome (Adriaenssens et al., 2018; Boros et al., 2018; Krishnamurthy, Wang, 2018).

The identification of atypical PBV-like sequences with a non-standard mitochondrial genetic code of fungi and invertebrates can serve as evidence that PBVs are capable of reproducing their own kind in the cells of lower eukaryotes, undergoing genetic changes when changing hosts (Yinda et al., 2018; Kleymann et al., 2020; Ghosh, Malik, 2021).

Therefore, it can be assumed that PBVs can get into eukaryotic cell (whether it is a cell of a fungus, an invertebrate or another host), where they will meet eukaryotic or prokaryotic virus with similar genome. Viruses like mitoviruses, partitiviruses, or cystoviruses may be among the PBV reassortment partners. After rearranging the genome segments of the PBVs with partner viruses, PBV-like reassortants described by Yinda et al. (2018) and Kleymann et al. (2020) can apppear. Then, according to the assumptions of the authors of the reassortation interaction hypothesis, depending on the presence or absence of prokaryotic motives and motives characteristic of PBVs, as well as depending on the genetic code used by the genes of a new virus (standard or mitochondrial) will determine not only the degree of its relationship with PBV, but also its prokaryotic or eukaryotic nature.

The hypothesis explaining the formation of atypical PBV strains through the exchange of homologous genome segments between related viral families does not exclude the possibility of their appearance using satellite relationships between PBVs and helper viruses from 2nd or 4th branches of the phylogenetic tree. Viruses from the families Partitiviridaе, Totiviridae, Reoviridae are known as helper viruses, which require some satellite dsRNAs for their reproduction (Lvov et al., 2013). Assuming that PBV is a satellite that uses an RdRp enzyme for reproduction in the fungal cells similar to mitovirus (from branch 2), it is possible to explain the appearance of non-capsid PBV-like strains with mitochondrial genetic code P11-300, P11-378, P14-90 and P15-218, isolated by Yinda et al. (2018) or M17A isolated by Kleymann et al. (2020).

By using as a helper a virus from branches 2 or 4 (similar to Partitiviridaе, Reoviridae, Totiviridae or Cystoviridae) the appearance of the encapsulated PBV-like strain P16-366 can be explained. In other words, in this case, the ability of the progeny resulting from the interaction of the satellite virus with the helper virus to reproduce in a bacterial cell or in the mitochondria of mold will depend on the nature of the helper virus. And this does not contradict the supposed prokaryotic nature of PBVs, but only means the possibility of its change in the reassortment process.

## Arguments in support of the hypothesis that PBV belongs to prokaryotic viruses

PBVs are found wherever bacteria are found – in environmental samples, in lower eukaryotes (fungi and invertebrates), in the gut contents of higher eukaryotes (including reptiles). This means that PBVs may not be intracellular eukaryotic viruses but prokaryotic viruses of the gut microbiome (Ghosh, Malik, 2021).

Like prokaryotic cystoviruses, PBV genomes contain and preserve in a saturated state (in both genome segments in each reading frame) functional binding sites for bacterial-type ribosomes (Shine–Dalgarno sequences), while in eukaryotic viruses the genome is not saturated with them (Boros et al., 2018; Krishnamurthy, Wang, 2018).

The identification of related PBV strains in different animal species may mean that the PBV hosts are a specific type of bacteria, possibly Firmicutes, found in the gut of different vertebrates and invertebrates. The level of preservation of prokaryotic sites in the genome of these bacteria (more than 80 % of the genes) corresponds to that of PBVs (Krishnamurthy, Wang, 2018).

PBV identification in the gut, respiratory organs of animals (cattle, humans, monkeys, pigs), in blood and respiratory samples (Lee, Bent, 2014; Blanco-Picazo et al., 2020) does not refute the assumption that bacterial cells can be their hosts. Phages cannot directly infect cells of different organs of higher eukaryotes, but they can get into these organs by non-specific translocation through gut epithelium with blood flow (Nguyen et al., 2017) or with the help of bacteria in which they multiply (Dabrowska et al., 2005). This is how phages penetrate into the blood, lymph, organs and even the brain.

The presence of a capsid protein with perforating activity (the ability to penetrate through the cell membrane), as in viruses capable of infecting animal cells (Duquerroy et al., 2009), does not contradict the fact that PBVs can be prokaryotic RNA viruses. It is known that representatives of the family of bacterial RNA viruses also have a molecular apparatus for penetrating into bacterial cells using transcytosis (Reed et al., 2013; Nguyen et al., 2017).

The acquisition of immunity to PBVs by infected animals also does not mean that PBVs can be considered eukaryotic viruses, since it has been established that host immune responses can also occur against bacterial viruses (Dabrowska et al., 2005; Górski et al., 2006). Possibly, PBVs cause an immune response to infection not of the host cells, but of the bacterial cells that make up its microbiome, which does not exclude the possibility that PBVs are prokaryotic viruses

Like the phages, which are virulent and moderate, in an infected organism, PBVs can be active (excreted) and inactive (temporarily not excreted), while infected animals will be asymptomatic carriers (Ganesh et al., 2014; Malik et al., 2014; Kumar et al., 2020; Ghosh, Malik, 2021).

The identification of PBV-like strains with the RdRp gene using an alternative mitochondrial genetic code of eukaryotes (mold, invertebrates) for translation is also not a refutation of the assumption that PBVs belong to prokaryotic viruses. According to the hypothesis of Wolf et al. (2018), they are the result of reassortment between the PBVs and the families of RNA viruses with a similar genome. The appearance of atypical PBV-like strains only indicates the possibility of an evolutionary transition from the prokaryotic nature of the virus to the eukaryotic nature and back as a result of rearrangement of genomic segments, and is explained by a change in the degree of saturation of the genome of the new virus with prokaryotic regions, which may change its nature. The possibility of satellite relationships between PBVs and RNA viruses of lower eukaryotes such as Partitiviridaе, Totiviridae and Reoviridae, which are known as helper viruses, is not excluded. There are known cases of satellite relationships between prokaryotic and lower eukaryotic viruses infecting one single-celled host, allowing phages to reproduce new progeny in a eukaryotic cell (Gogarten, Townsend, 2005; Claverie, Abergel, 2009; Thannesberger et al., 2017).

The expression and functioning of PBV segment 1 in vivo in Escherichia coli (Boros et al., 2018) was carried out, which confirms the existence of a bacterial culture for the propagation of PBVs.

## Conclusion

The arguments given convincingly show that PBVs can indeed be prokaryotic viruses, since they are found wherever bacteria are found – in environmental samples, in the gut contents of vertebrates, in the cells of fungi and invertebrates.

Similar to prokaryotic cystoviruses, PBV genomes contain and retain functional binding sites for bacterial-type ribosomes in a saturated state, while in eukaryotic viruses the genome is not saturated with these motifs. The high level of preservation of prokaryotic sites in the PBV genome suggests that they belong to a new family of RNA viruses that infect a certain type of bacteria with a high content of SD-sequences in the genome. Such host bacteria of PBVs can be Firmicutes found in the gut of vertebrates and invertebrates, which have a similar level of prokaryotic motifs characteristic of bacteria. The high frequency of the presence of SD-sequences in the PBV genome can be considered one of the main criteria for identifying new virus families for affiliation to a prokaryotic or eukaryotic host.

But in order for a virus to be finally classified as a phage, its genome must contain a structural gene encoding a protein with specific proteolytic properties that determine the ability of the virus to independently penetrate into a bacterial cell. A protein with proteolytic properties is present in the PBV capsid, which indicates the ability of this virus for horizontal transmission (independently) from cell to cell and from one host to another. At the same time, the identification of PBVs in the gut of various hosts may suggest that their horizontal transmission can be carried out by bacteria and, therefore, these viruses themselves are phages. The identification of PBVs in various tissues of the body can also be explained by the fact that, being phages, they are capable of nonspecific translocation through the walls of gut epithelial cells.

The selection of a culture for the virus propagation is necessary for the final determination of its nature. To date, the belonging of PBVs to viruses of higher eukaryotes has not been proven, since it was not possible to isolate them from eukaryotic cell cultures. To establish definitively whether PBVs are prokaryotic viruses, it is necessary to direct efforts to select a host for their reproduction among prokaryotic cells obtained from the gut microbiome of mutants and conditions for the cultivation of these cells.

And, finally, the presence of probable relatives among the families of RNA viruses with similar genes with which PBVs can exchange genome segments, replicating atypical genetic variants, does not contradict the correctness of the hypothesis about the phage nature of PBVs, but indicates their potential ability to adapt to new conditions of existence, allowing them to infect eukaryotic or prokaryotic host cells.

## Conflict of interest

The authors declare no conflict of interest.
